# Free radical detection in precision-cut mouse liver slices with diamond-based quantum sensing

**DOI:** 10.1073/pnas.2317921121

**Published:** 2024-10-14

**Authors:** Yue Zhang, Alina Sigaeva, Arturo Elías-Llumbet, Siyu Fan, Willem Woudstra, Rinse de Boer, Elkin Escobar, Claudia Reyes-San-Martin, Robin Kisabacak, Dorenda Oosterhuis, Alan R. Gorter, Britt Coenen, Felipe P. Perona Martinez, Geert van den Bogaart, Peter Olinga, Romana Schirhagl

**Affiliations:** ^a^Department of Biomaterials and Biotechnology, University of Groningen, University Medical Center Groningen, Groningen 9713 AV, The Netherlands; ^b^Laboratory of Genomic of Germ Cells, Biomedical Sciences Institute, Faculty of Medicine, University of Chile, Independencia Santiago 1027, Chile; ^c^Department of Molecular Immunology, Groningen Biomolecular Sciences and Biotechnology Institute, University of Groningen, Groningen 9747 AG, The Netherlands; ^d^Molecular Genetics Group, Max Planck Tandem Group in Nanobioengineering, Faculty of Natural and Exacts Sciences, University of Antioquia, Medellin 1226, Colombia

**Keywords:** diamonds, quantum sensing, NV center, nanodiamonds

## Abstract

Free radical generation plays a key role in many biological processes including cell communication, maturation, and aging. In addition, free radical generation is usually elevated in cells under stress as is the case for many different pathological conditions. In liver tissue, cells produce radicals when exposed to toxic substances but also, for instance, in cancer, alcoholic liver disease and liver cirrhosis. However, free radicals are small, short-lived, and occur in low abundance making them challenging to detect and especially to time resolve, leading to a lack of nanoscale information. Recently, our group has demonstrated that diamond-based quantum sensing offers a solution to measure free radical generation in single living cells. The method is based on defects in diamonds, the so-called nitrogen-vacancy centers, which change their optical properties based on their magnetic surrounding. As a result, this technique reveals magnetic resonance signals by optical means offering high sensitivity. However, compared to cells, there are several challenges that we resolved here: Tissues are more fragile, have a higher background fluorescence, have less particle uptake, and do not adhere to microscopy slides. Here, we overcame those challenges and adapted the method to perform measurements in living tissues. More specifically, we used precision-cut liver slices and were able to detect free radical generation during a stress response to ethanol, as well as the reduction in the radical load after adding an antioxidant.

## Significance

Here, we show diamond-based quantum sensing in tissue slices. We demonstrated this in precision cut liver slices which can be maintained alive for several days. This method allows measurements of free radical generation at the nanoscale. We were able to show that we can measure free radical generation during a stress response as well as after adding ascorbic acid.

Diamond magnetometry is a powerful quantum sensing technique that allows to read out magnetic resonance signals in the nanoscale (1). This technique offers local nanoscale measurements with high sensitivity and resolution (2, 3). In physics, it has already been successfully used to measure the magnetic signature of hard drives (4), magnetic nanostructures (5) as well as magnetic domains (6). In biological applications, diamond magnetometry has been useful in measuring magnetic particles in bacteria (7), spin labels (8) as well as labeled slices of fixed cells (9). One specific mode of diamond magnetometry called relaxometry is particularly attractive since it is relatively straightforward and does not require microwave radiation (10). In a typical relaxometry (or T1) measurement, nitrogen-vacancy (NV) centers are brought into a bright ms = 0 state by laser pulses. Then, the amount of NV centers that are still in the bright state is measured after varying dark times. The time it takes the NV centers to relax back to the equilibrium state is shortened by magnetic noise. Also, this method has been used already for several applications. These include measurements of paramagnetic ions (11), spin diffusion (12), and, in combination with responsive coatings, of pH, redox potential and viruses (13, 14). Most recently, free radical generation in living cells has been demonstrated (15–18). These radicals are characterized by free electrons, which cause a shortening in relaxation time. Free radicals play a key role in many biological processes including cell maturation, cell communication as well as aging. In addition, free radical generation is usually elevated when cells are under stress, which is the case for many diseases including, for example, cancer, cardiovascular diseases as well as bacterial or viral infections. While relaxometry has been used in several different cell types including yeast (19), immune cells (20), bacteria (21), and sperm cells (22), the technique has not been used for tissues yet.

Here, we demonstrate relaxometry in tissues. More specifically, we performed measurements on precision-cut tissue slices as a model (23). Such precision-cut organ slices are viable for several days and can be used to study cells in their natural environment (24). Precision-cut organ slices are an attractive tool to reduce the number of animals in animal experiments for several reasons (25). First, it is possible to obtain several slices from an organ or a wedge biopsy. Additionally, it is attractive to use precision-cut organ slices from humans to reduce differences between animal and human experiments (26).

**Fig. 1.**
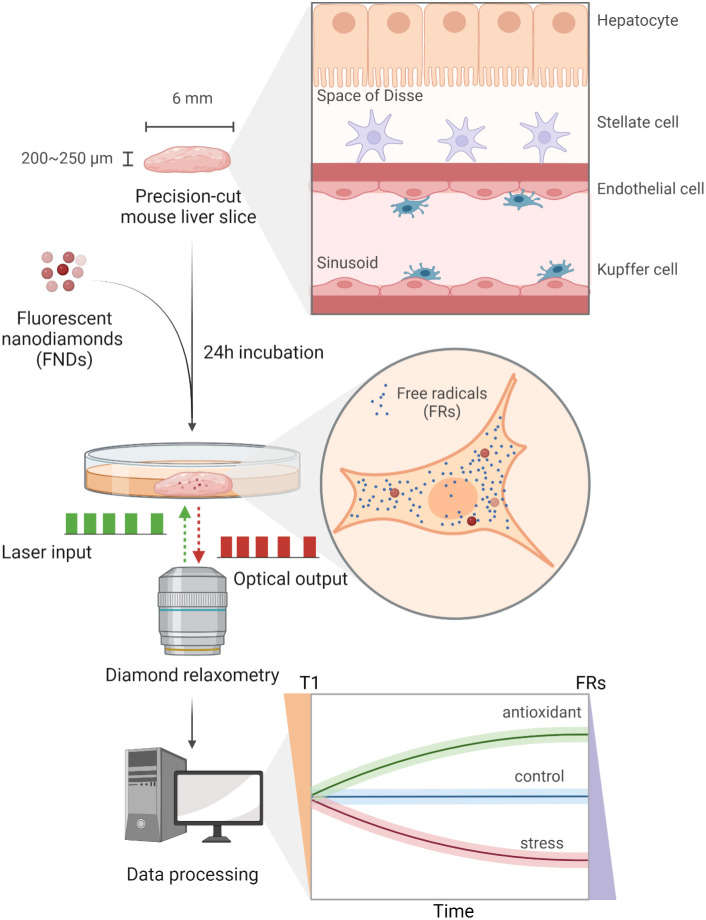
Free radical detection in mouse liver slices with diamond relaxometry. Precision-cut mouse liver slices maintain the diversity and characteristics of multicellular components in organs. FNDs were incorporated into liver slices during the 24 h incubation. In diamond relaxometry, a sequence of green laser pulses was applied to excite FNDs and optical readouts were collected afterward. After data processing, the evolution of T1 indicates the dynamics of free radicals in the presence of different treatments. An increased level of free radicals results in reduced T1 values, and vice versa. The figure was created with BioRender.

Here, we used precision-cut slices from the liver to demonstrate free radical sensing by relaxometry (Fig. 1). We investigated the viability of liver slices after fluorescent nanodiamonds (FNDs) uptake. We investigate the distribution of FNDs in the liver slices after incubation. Additionally, we outline the technical challenges and points of consideration, providing handholds for any future studies that aim to use nanodiamond relaxometry in live tissue samples.

## Materials and Methods

### Animal.

Adult male C57BL/6J mice aged 13 to 18 wk or 80 wk were obtained from Harlan Laboratories B.V and Charles River Europe. Animals were kept in a temperature and humidity-controlled room with a 12 h light/dark cycle with food and water ad libitum. Mice were allowed to acclimatize for at least 1 wk before we started the experiments. Mice were killed under isoflurane/O_2_ anesthesia and their livers were harvested. The organ was kept in an ice-cold University of Wisconsin (UW) organ preservation solution (DuPont Critical Care) until the start of the slicing. All experiments were conducted according to Dutch law and approved by the Institutional Animal Care and Use Committee of the University of Groningen and the Central Committee on Animal Experiments (permit number AVD10500202215969).

### Precision-Cut Liver Slices (PCLS).

PCLS were prepared as described previously (27, 28). Briefly, mouse liver cores were obtained by using a biopsy puncher with a diameter of 6 mm. The cores were then sliced using a Krumdieck Tissue Slicer MD6000 (Alabama R&D), which was filled with ice-cold Krebs–Henseleit buffer. The buffer was supplemented with 25 mM D-glucose (Merck), 25 mM NaHCO_3_ (Merck), and 10 mM HEPES (MP Biomedicals) and saturated with a mixture of 80% O_2_ and 5% CO_2_. Following the slicing procedure, liver slices with a thickness of approximately 200 to 250 µm and a wet weight of about 5 mg were preserved in the UW organ preservation solution on ice until further use.

### Tissue Uptake of FNDs.

We used 70-nm and 120-nm fluorescent high-pressure high-temperature nanodiamonds (FNDs, Adamas Nanotechnologies). The FND suspension in water (1 mg/mL) was sonicated for 10 min and then diluted in the complete medium used for the slice incubation on the day of the slicing to a final concentration of 50 µg/mL or 20 µg/mL

The mouse liver slices were cultured in William’s E Medium (GlutaMAX™ Supplement, Invitrogen) supplemented with 25 mM D-glucose and 50 µg mL^−1^ gentamycin (Invitrogen) (the complete medium is denoted as WEGG and simply referred to as medium) at 37 °C, using a gas mixture composed of 80% O_2_ and 5% CO_2_ (referred to as gas mixture). The slice culture incubator was positioned on a horizontal shaking table set at 70 rpm. The liver slices were placed into individual wells of a 24-well plate. Each well contained 500 µL of prewarmed (37 °C) and presaturated (gas mixture) medium supplemented with FNDs at desired final concentrations. After 24 h incubation, the mouse liver slices were rinsed with fresh medium to remove free particles and ready for the following experiments.

### Two-Photon Microscopy.

The images were acquired with Zeiss 780 two-photon confocal microscope. The mouse liver slice was immobilized on the glass bottom petri dish and excited with 850 nm laser. The signals were obtained in Lambda mode from 410 nm to 690 nm. The spectrum was divided into 32 channels and display rainbow color code from purple (410 nm) to red (690 nm). The characteristic emission range of FNDs is above 630 nm and indicated in red. The majority of autofluorescence from tissue is below 550 nm and shows green overall. The XY axis resolution is 220 nm and Z axis resolution is 1 µm. 3D image and video were processed with Imaris.

### Immunostaining on Cryosections.

Four µm cryosections of liver slices were cut with Cryostar NX70 cryostat (Thermo Fisher Scientific) and collected with adhesive glass slide (StarFrost^®^). Sections were dried under the fan and fixed with acetone for 10 min at the room temperature (RT). The samples were blocked with 3% bovine serum albumin (BSA) in phosphate buffer saline (PBS) for 30 min at the RT to reduce the nonspecific binding. Then, the sections were incubated with primary antibody [diluted in PBS containing 5% fetal bovine serum (FBS) (5% PBS-F)] for 1 h followed by staining with secondary antibody (diluted in 5% PBS-F) for 45 min in the dark at RT. Afterward, nuclei were stained with 4 µg/mL DAPI for 5 min in the dark. Next, the samples were incubated with Sudan black (1 mg/mL in 70% ethanol) for 20 min in the dark to minimize the autofluorescence. Finally, the slices were mounted (ProLong™ Diamond Antifade Mountant, Thermo Fisher) and kept at 4 °C until imaging. A purified rat anti-mouse CD31 primary antibody (BD, 1:100 dilution) was used to label endothelial cells. A rat anti-mouse CD68 monoclonal antibody (BIO-RAD, 1:100 dilution) was used to label Kupffer cells. The secondary antibody was goat anti-rat Alexa Fluor™ 488 (Thermo Fisher Scientific, 1:400) for both conditions.

Fluorescent images were acquired with Leica SP8X confocal microscope using 40× objective lens. FNDs were exited with a 561 nm laser, emissions were collected from 680 nm to 750 nm, Alexa Fluor 488 was exited with 488 nm laser, and emissions were collected from 505 nm to 549 nm. The XY pixel size is 220 nm. The whole image is 228 µm by 228 µm.

### Electron Microscopy.

The embedding procedure was done similarly as previously reported (29). The mouse liver slice was fixed with 2% glutaraldehyde and 0.5% paraformaldehyde in 0.1 M cacodylate buffer in a glass vial. Then, the sample was stained with 1% osmium tetroxide/1.5% potassium ferrocyanide for more than 2 h at 4 °C followed by rinse with ultrapure water. Afterward, the slice was dehydrated in an increasing graded ethanol series (30%, 50%, 70%, and 100%) and the ethanol was replaced with acetone, epoxide resin (EPON)/acetone mixture (1:3, 1:1, and 3:1) and pure EPON step by step. Finally, EPON was polymerized overnight at 58 °C. Areas containing tissues were selected using a stereo microscope and sawn from the EPON block. Subsequently, 200 nm sections were collected on formvar-coated carbon evaporated copper grids. Sections were analyzed using a CM12 transmission electron microscope (Philips) running at 100 kV. For electron tomography, sections were decorated with 10-nm gold particles and dual tilt series were recorded manually from −40° to 40° with 2.5° increments. Tomograms were reconstructed and combined using back projection in the Imod software package.

### Tissue Viability: ATP/Protein Assay.

The tissue viability was assessed by measuring the ATP content normalized to the protein content as previously described (23). The ATP assay was conducted using a bioluminescence kit (Roche Diagnostics, Mannheim, Germany). Briefly, the individual liver slice was collected in 1 mL of SONOP buffer (2 mM EDTA in 70% v/v ethanol at pH 10.9), snap-frozen in liquid nitrogen, and kept at −80 °C. Then, the samples were homogenized twice for 45 s with a Mini-BeadBeater 24 (Biospec Products) and centrifuged at 13,000 rpm for 5 min at 4 °C. To determine the ATP content, the supernatant was diluted 10-fold in a 0.1 M Tris HCl buffer (pH 7.8) containing 2 mM EDTA. The diluted samples and a standard curve were transferred to a white 96-well plate, ATP reaction mix was added and luminescence was measured after 5 min using a Synergy HT plate reader (Biotek).

The pellets after centrifugation were dried at 37 °C for 24 h and obtained for a Lowry assay (BIO-RAD, Protein Assay, USA). The samples were dissolved in 200 µL 5 M NaOH for 30 min at 37 °C and then diluted with 800 µL ultrapure water, and homogenized for 40 s with a Mini-BeadBeater 24. The supernatant and BSA standard curve were transferred to a transparent 96-well plate, assay reaction mix was added, and after 15 min, the absorbance was measured at 650 nm with a Synergy HT plate reader.

Serial concentrations of ATP-standard and bovine serum albumin were prepared as calibration curves for quantification. Three slices were collected for each condition and slices not exposed to FNDs were used as control.

### FACS for Determining the Amount of Cells Which Contain FNDs.

To determine how many cells contain FNDs, we disintegrated and FACS sorted and imaged the cells. More specifically, after 24 h of incubation with FNDs, the tissue slices were collected and a single-cell suspension was derived by mechanical transfer through a 100-μm cell strainer (Greiner Bio-One). Cells were washed in PBS twice and fixed in 4% PFA. Cells were sorted using a MoFlo Astrios Cell Sorter (Beckman Coulter) and deposited on adhesion microscope slides (Marienfeld).

After sorting, cells were counterstained with phalloidin-FITC and DAPI to visualize the cell borders and the nuclei. The samples were mounted with ProLong™ Diamond Antifade Mountant (Invitrogen) and stored at +4 °C until imaging.

The imaging was done with Leica SP8X or Zeiss LSM800 Airyscan confocal laser scanning microscope. We collected z-stack images of at least 100 individual cells in each cell population, recording the fluorescence of DAPI, phalloidin-FITC, and FNDs, as well as transmitted light (DIC). We then calculated the percentage of cells in each cell population that contained at least one FND in their cytoplasm.

### Immobilization of the Slices for Relaxometry.

A stainless-steel anchor was designed (*SI Appendix*, Fig. S1) and produced to immobilize the slices. The metal threads and notches on the wall ensure sufficient media exchange. The anchor was sterilized with 70% ethanol before use. One slice was transferred to 2 mL prewarmed medium in one compartment 35 mm glass bottom Petri dish. The anchor was placed on the slice gently and the dish was moved to the T1 setup with a transportation box prefilled with the gas mixture.

### Nanodiamond Relaxometry.

Nanodiamond relaxometry was performed in tissue slices incubated with FNDs for 24 h, using a custom-built setup. The relaxometry setup has been described previously (15, 16, 19). It is based on a confocal microscope, where a green laser (Torus 532 nm, Laser Quantum) is used to locate FNDs in the sample. The FND photoluminescence and tissue autofluorescence are collected through the same objective (Olympus UPLSAPO 100XO, Oil immersion, NA 1.40), filtered with a 550 nm long-pass dichroic mirror and an additional filter (600 nm long-pass, 650 nm long-pass, or 700 nm long-pass). The filtered light is collected by a photon-counting avalanche photodiode (Excelitas Technologies SPCM-AQRH) and can be used to obtain a confocal image of the sample. The fluorescent signal of FNDs in the far-red region is usually significantly higher than the autofluorescence of the sample and is not prone to bleaching. Thus, one can use the resulting image to locate FNDs inside the tissue sample.

As the laser can additionally be pulsed with very high precision, the same setup can be used to perform relaxometry measurements. Once the operator identifies an FND, the T1 pulsing sequence can be started. It consists of 5 µs laser pulses, separated by logarithmically increasing dark times τ (0.2 µs to 2 ms), when the laser is switched off. The energy per pulse was approximately 0.3 nJ. We used an AOM in a double-pass configuration to produce the pulses. Then, the laser is conducted to the microscope by an optical fiber. This relatively low laser power has been chosen to avoid photodamage to the tissue samples. In previous experiments, no detectable temperature increase due to the laser irradiation has been found when using this pulsing scheme (20). The laser pulses are used to first initialize and then read out the spin state of the NV centers in the FND after a given dark time τ. To obtain a single relaxation curve, the T1 sequence is repeated 10,000 times, which takes approximately 2 min. The photoluminescence is then summed, and the integrated signal over the first 1 µs of each laser pulse is considered the fluorescence intensity of the FND after a corresponding τ (PL(τ)). PL(τ) can then be plotted as a function of τ, and the resulting relaxation curve is fitted by a double exponential function, using a MATLAB script, to extract T1 (11, 30). The relaxometry measurements can then be performed on multiple different particles within one sample or between different samples to compare the free radical load in different surroundings (performed to compare young and old mice).

Alternatively, one can increase the number of repetitions, monitoring the photoluminescence of a single FND over longer experiments. In this paper, we tracked the changes in T1 of individual FNDs, as the slices were exposed to a trigger (25 mM ethanol, distilled water, or 0.6 mM L-ascorbic acid). Ethanol is a known trigger of free radical production in liver (31, 32), whereas L-ascorbic acid has been shown to decrease the free radical load (33). In these experiments, we first located an FND within a liver slice and recorded a preliminary T1 to assess the baseline free radical load in the close vicinity of the particle. Based on previous studies in cells (15, 16), we have selected the FNDs that showed the initial T1 values of 130 µs or higher to avoid the areas and cells with high baseline radical load (possibly due to the stress and/or death of the cells in the tissue slice due to the cutting procedure). These initial values served as the baseline reference for each individual FND. Then, we added the solution of one of the triggers to the medium, in which the liver slice was kept, and immediately started the pulsing sequence, recording the changes in FND fluorescence over the course of 33 min. The resulting dataset was then analyzed with the rolling window approach, described previously (16, 18). Briefly, first 50,000 repetitions (corresponding to minutes 0 to 10 of the experiment) are used to extract a single T1 value. Then, the summation window is shifted by 10,000 repetitions (corresponding to minutes 2 to 12 of the experiment), and the new subset is used to obtain the next T1 value. This process is repeated, until the summation window reaches the last data in the dataset. The resulting T1 values can then be plotted against the incubation time to show the evolution of the free radical load in the sample.

### Statistical Analysis.

Unpaired *t* test was applied to compare two groups in tissue viability. An ordinary one-way ANOVA with Holm–Sidak multiple comparisons test was used to compare T1 values between different locations within the same tissue slice. Simple linear regression was used to estimate the effect of the depth within the tissue slice on the recorded T1 values. A ratio paired *t* test was used to compare the T1 values before and after the treatment with a trigger. Statistical analysis was performed in GraphPad Prism 10. Levels of statistical significance were chosen as follows: *P < 0.05, **P < 0.01, ***P < 0.001, ****P < 0.0001.

## Results and Discussions

There are several relevant differences between cells and tissues that posed challenges for relaxometry measurements.

First, tissue slices are complex inhomogeneous systems. They consist of numerous cells of different types, as well as extracellular matrix. For relaxometry measurements, FNDs need to be in close vicinity of the source of free radicals. While FND uptake in cultured cells is relatively well described, much less is known about the timing and efficiency of uptake in tissue slices. Moreover, FNDs might have adverse effects on tissue viability.

Second, in tissue slices, cells have less easy access to nutrients and oxygen in the medium, as compared to cultured adherent cells. During the incubation of the slices, shaking and high oxygen levels are used to ensure that all cells have enough nutrients and oxygen to be viable (34). This approach would require major modifications of the existing relaxometry setup. As a result, it is more challenging to keep tissue slices viable during the measurement, which might manifest in higher levels of baseline cell stress. Stressed cells might show increased production of free radicals regardless of the intervention, which would interfere with the experiments. We have thus investigated the possible impact of suboptimal environment on the slice viability.

Third, while cells adhere to the bottom of Petri dishes and are generally not moving a lot, this is not the case for tissue slices. Therefore, we had to ensure the stable position of the slices without damaging them.

Finally, tissues have strong background fluorescence due to the thickness of the sample. This obstacle was addressed by introducing a different filter to collect fluorescence of the NV centers.

### FND Uptake and Location.

**Fig. 2.**
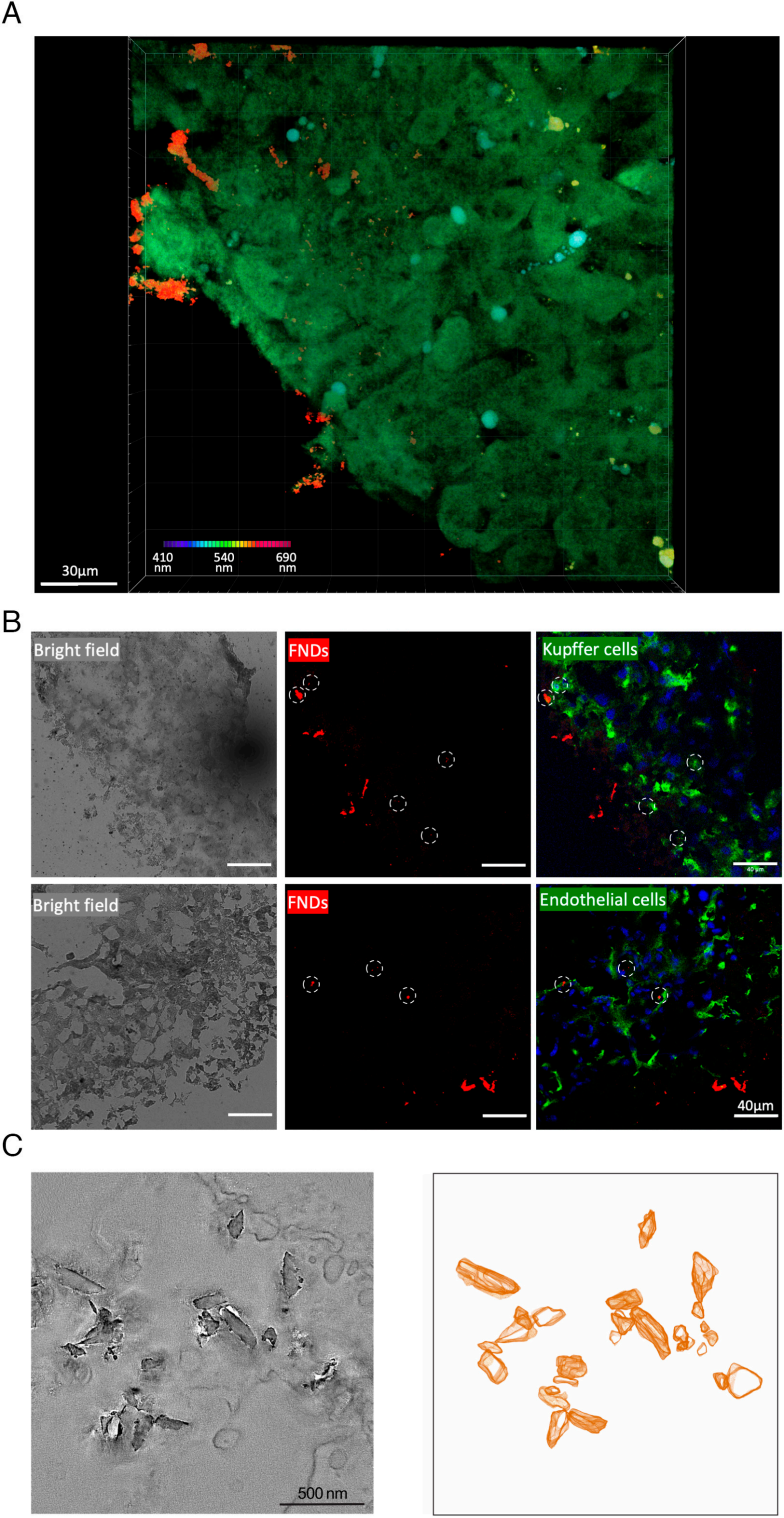
Nanodiamonds in precision-cut mouse liver slices. (*A*) two-photon microscopy on mouse liver slice. The image was acquired with lambda mode and each color represents a different emission band from 410 nm (purple) to 690 nm (red). Red signals are FNDs and green signals are tissue autofluorescence. (*B*) Immunostaining on 4 µm cryo-sections. Grey: bright field; Green: CD68 antibody-labeled Kupffer cells or CD31 antibody-labeled endothelial cells; Red: FNDs, some FNDs were indicated with a circle; Blue: DAPI-labeled nucleus. (*C*) Electron microscopy images of FNDs in the tissue: Tomographic slices (*Left*) and 3D-rendered volume of FNDs (*Right*).

A requirement for diamond-based quantum sensing is that the NV centers need to be close to the source of magnetic noise. In general, the sensitivity decreases with the distance r^6^. In practice, this means that the NV centers need to be within some tens of nm from the free radicals. To achieve that, FNDs need to penetrate into the tissue slices. Conventional confocal microscopy requires that the specimen is very thin while two-photon microscopy can reach up to 1 mm depth in the tissue and therefore is ideal for liver slices (35). With a fixed excitation laser, we chose lambda mode for the scanning, and each color indicates a different emission band. FNDs show red fluorescence while autofluorescence from tissue is typically green. It is evident from the 3D two-photon microscopy picture in Fig. 2*A* that FNDs can be found within the tissue slice. More details can be found in Movie S1.

Immunofluorescent images, shown in Fig. 2*B* shine further light on the exact location of FNDs within the tissue. Generally, besides connective tissue, the liver contains four major cell types, i.e., hepatocytes, hepatic stellate cells, Kupffer cells, and liver sinusoidal endothelial cells (36). We chose to stain for Kupffer cells and liver sinusoidal endothelial cells since these cell types have been reported to be mainly responsible for particle uptake in liver cells (37, 38). Especially Kupffer cells are known to ingest large numbers of nanoparticles since these cells are the macrophages of the liver. As a result, they are known to actively ingest different kinds of nanoparticles (39). Bartucci et al. for instance found that Kupffer cells ingest polystyrene particles (23). Dragoni et al. reported all major cell types in the liver contributed to particle uptake. However, Kupffer cells were the most important cells for gold nanoparticle uptake (40). Similarly, we find FNDs within both Kupffer cells and endothelial cells. Besides that, we also found particles between cells as well as at the border of the slices.

To further confirm these findings, we performed transmission electron microscopy of 200-nm-thick slices produced from the PCLS (Fig. 2*C*). We found several FND clusters but also single NDs in cells as well as in the intercellular space within the tissue. More details can be found in Movie S2.

To determine which percentage of cells contained FNDs, we disintegrated the tissue, FACS sorted, and imaged the cells. During this procedure, we found two populations of cells which differed slightly in terms of their autofluorescence. The overall percentage of cells with FNDs was 34% (33% in the highly fluorescent group and 35% in the low fluorescence group). Since these numbers are for the whole tissue, we would expect the numbers closer to the surface (where we perform our measurements) to be higher (*SI Appendix*, Fig. S3).

### Viability with FNDs.

**Fig. 3.**
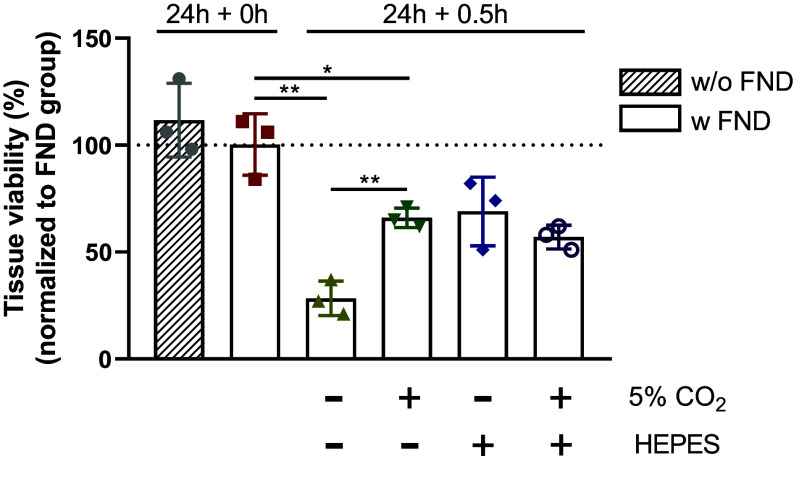
Tissue viability of mouse liver slices in different conditions. PCLS were incubated with or without FNDs for 24 h at 37 °C, 80% O_2_ and 5% CO_2_. After that, different approaches were applied to maintain tissue viability after slices were removed from slice culture incubator for 0.5 h. One way is that PCLS were transferred to a 5% CO_2_/ambient O_2_ incubator for 0.5 h. Another way is to keep PCLS in WEGG supplemented with 10 mM HEPES buffer at 37 °C with ambient environment. The third way is to combine two methods. All data were normalized to FND group (100%). Unpaired *t* test was applied to compare between two groups. The whiskers are shown as mean with SD, *P < 0.05, **P < 0.01, ***P < 0.001, ****P < 0.0001.

Another challenge that we encountered is that PCLS are more fragile than cells. Compared to cells, tissue slices do not obtain nutrients via surrounding medium as easily. Generally, the constant horizontal shaking of the incubator is needed for efficient medium and air exchange. Besides, WEGG medium has no capacity to maintain the pH under ambient CO_2_ pressure. We observed a quick change in pH from 7.4 (5% CO_2_) to above 8.5 (ambient air) in minutes (*SI Appendix*, Fig. S2) in ambient environment. Last, liver slices are cultured at high oxygen concentration (80%) and higher temperature (+37 °C), as compared to the ambient conditions. These changes might affect the viability of the slices, which might necessitate the use of an incubation unit during the T1 measurements. To assess the severity of the problem and to optimize the tissue handling, we performed a viability assay before and after the different experimental steps (see Fig. 3, first three columns).

For most assays, it is problematic that the number of cells in tissue slices cannot be determined as accurately as in cell cultures due to anatomical variations. To account for this, we chose to perform an ATP/protein assay. This assay, which has been established earlier, measures ATP content in relation to the overall protein levels (23). ATP levels were affected by both cell viability and cell numbers while protein level was only influenced by cell numbers. This allows us to compensate for the fact that different slices vary in cell number. During our initial tests, we observed a drastic drop in viability after the tissues were exposed to WEGG medium and ambient air at 37 °C for only 30 min. We can also see that there is no difference between samples with and without FNDs. This indicates that the measurement conditions rather than the FNDs themselves are problematic. This was expected since the literature shows excellent biocompatibility of FNDs with different cell types as well as in vivo (41, 42). Also, biocompatibility in the liver was shown in rats as well as in hepatocytes (43).

To resolve the problem of tissue viability loss and to counteract the pH change, we have applied two strategies: using HEPES buffer and measuring in a 5% CO_2_ environment. Both methods are known to buffer against an increasing pH (44). Fig. 3 (last three columns) shows the viability assessment after adaptation of the protocol. When comparing to initial conditions (ambient air), the viability of slices cultured with HEPES, 5% CO_2_, or both led to a significant improvement from around 30 to 70% in all cases. Such an improvement in viability has also been observed in literature and was thus expected (45). When comparing the usage of 5% CO_2_ and HEPES alone with using both, we do not see a further improvement. For this reason and since HEPES has been reported to induce free radical generation (46), we have decided to use 5% CO_2_ for the rest of the study.

### Different Approaches for Slice Immobilization.

Our homemade T1 setup is based on an optical detection system and adapted from an inverted confocal microscope. Due to the limitation of the working distance of the objective lens, the sample needs to be close enough to the glass surface. Unlike adherent cells, mouse liver slices are normally suspended in media and hence need to be restrained during the measurement. Besides, free radical detection with diamond relaxometry requires living tissue and the slices need to be treated carefully.

**Fig. 4.**
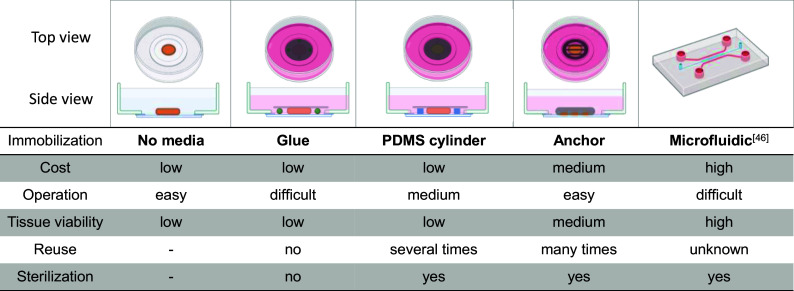
Summary of different approaches to immobilize liver slices.

Several different ways were explored to immobilize the slices and summarized as shown in Fig. 4. The first and simplest way is to remove as much medium as possible. At the cost of viability, we can conduct T1 measurements, but this approach is not feasible for free radical detection because free radicals would also be generated under control conditions in this case. Next, we were focusing on building a small cage with a cover glass and glue. The liver slice was put at the center of the Petri dish and several tiny drops of biocompatible glue were added to its surroundings without direct contact. Then, the cover glass was put on the slice with caution until fixed with glue. At last, fresh medium was added to the dish. However, during the preparation, the liquid glue is hard to control and can spread to the slice. The exposure of medium to the glue also poses a risk for tissue viability. Therefore, poly(dimethylsiloxane) (PDMS) cylinders with a specific height (around 250 µm) were made to replace the glue. With this approach, the space left for slices can be well controlled. PDMS cylinders also can be collected afterward and sterilized with 70% ethanol several times until deformation. Nevertheless, the tissue viability still drops quickly due to the cover glasses impeding the free exchange of air and nutrients in the tissue. We also observed that some slices attached to the cover glass afterward due to the surface tension and partly fallen apart.

Given the above, we designed and developed stainless-steel anchors for this purpose. There are several advantages of this design. The material is inert and can be easily sterilized with ethanol before use. The metal threads and notches on the wall ensure sufficient nutrient exchange. Besides, the distance between threads and the bottom is optimized for single slices without pressure. Finally, the anchor is user-friendly and can be easily placed and removed with a tweezer. We performed diamond relaxometry with the metal anchor and 5% CO_2_ supply at 37 °C. The anchor also can be produced by 3D printing in the cases when the biocompatibility and density of materials should be taken into consideration (for example, with long-term experiments).

For long-term experiments, more sophisticated devices such as perfusion chambers (47) and microfluidics/microchambers might also be required (48). Those devices are closed systems to avoid evaporation of the culture medium and ensure precise control over the environmental variables including temperature, pH, or gas mixture level.

### Signal-to-Noise Ratio (SNR) and T1.

**Fig. 5.**
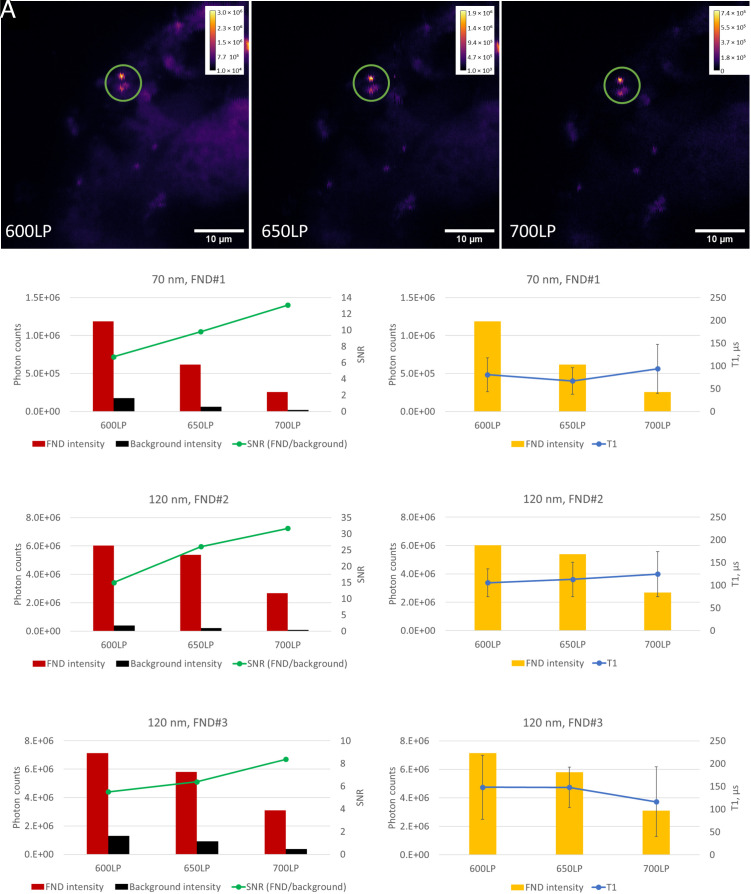
The effect of filter wavelength on the image and signal quality. (*A*) The same area of a liver slice with internalized FNDs was imaged with different filters (600 nm long-pass, 650 nm long-pass, or 700 nm long-pass). As the overall signal intensity drops, the internalized FNDs (green circle) become more apparent. Calibration bars show fluorescence intensity in photon counts integrated over 1 ms. (*B*) Comparison of the fluorescence intensity of FNDs, tissue autofluorescence, and recorded T1 values under different filter wavelengths for three individual FNDs. FND#1 comes from a batch of smaller FNDs with an average hydrodynamic diameter of 70 nm, whereas FND#2 and FND#3 have a size of 120 nm, like FNDs used in the rest of the study. Red bars and yellow bars show the fluorescence intensity of the FND and black bars the average intensity of the tissue autofluorescence. The green line shows the SNR, defined in this case as the ratio between the FND fluorescence intensity and the tissue autofluorescence intensity. The blue line indicates the T1 values recorded from the same FND under different filter settings. The error bars show 95% CI for the calculated T1 values.

As with all optical microscopy techniques, the strong autofluorescence of tissue components such as flavoproteins becomes problematic due to the thickness of the sample. Here, it has to be noted that tissue autofluorescence is less problematic for T1 measurements since FND fluorescence does not photobleach. While in conventional optical imaging, one needs to measure right away to avoid bleaching of organic dyes, T1 measurements can be conducted after (some of) the autofluorescence has photobleached. This approach has been used before to image FNDs with excellent SNR after photobleaching (49). However, during our measurements, FNDs are constantly moving to different regions and have to be tracked, which renders this approach less useful in our case. Moreover, effective photobleaching might cause a local increase in free radical concentration due to the transfer of electrons to molecular oxygen, affecting both the sample health and the results of the measurements. To evaluate the problem and to optimize the measurement conditions, we first acquired confocal images of the tissues (shown in Fig. 5). In cells and for applications in physics, NV center fluorescence is typically collected with a long-pass filter above 600 nm (50). This wavelength is chosen to maximize the fluorescence collection from NV centers (emitting a broad peak between 600 nm and 800 nm) (51). However, the spectral range where the autofluorescence is lowest is at a higher wavelength. Spectra of the NV centers as well as our tissues are shown in *SI Appendix*, Fig. S4. Following this rationale, we increased the wavelength above which we collect the NV center fluorescence to 650 nm or 700 nm. Already from the confocal images shown in Fig. 5, it is evident that increasing the filter wavelength improves image quality.

Further quantitative analysis (Fig. 5) confirms that the SNR indeed improved significantly with the filter wavelength. While we reduce the amount of light that we can collect from NV centers by discarding the lower wavelength portion of the signal, we remove more of the background.

To rule out whether there is an influence on the T1 values that we measure, we have also compared the T1 values that we calculated from the curves integrated over the same duration for the different filter settings. As expected, we did not see any significant differences between T1 values for the different filter settings. This means that we do not introduce any errors by applying this method. At the same time, different filters allow us to calculate T1 values with different level of confidence (Table 1).

**Table 1.** 95% CIs for the T1 values shown in Fig. 5

Filter used to collect FND fluorescence	FND#1 (70 nm)	FND#2 (120 nm)	FND#3 (120 nm)
600 nm long-pass	±38%	±29%	±47%
650 nm long-pass	±36%	±34%	±30%
700 nm long-pass	±48%	±40%	±66%

Relatively short-wavelength filter (600LP) allows one to collect the largest portion of fluorescence, including the autofluorescence of the tissue. Moreover, we record fluorescence of all NV centers, both those in neutral (NV^0^) and those in negative (NV^−^) charge state. Emission spectrum of NV^0^ centers is shifted to the shorter wavelength range (575 nm to 700 nm), compared to the NV^-^ fluorescence (640 nm to 750 nm) (52). Only NV^-^ centers can be used for quantum sensing, which calls for using longer collection wavelengths (16).

At the same time, as a longer collection wavelength decreases the total amount of fluorescence collected from a single FND, the precision of the measurement is also decreased (Table 1). In practice, this reduces the temporal resolution of the technique, as one would need to integrate the signal over longer time periods to obtain less noisy T1 relaxation curves. This might become particularly problematic if smaller and/or less bright FNDs are used for the experiments. For this reason, we used a 650-nm long pass filter to collect NV fluorescence in the following measurements.

### T1s at Different Areas of the Slice.

**Fig. 6.**
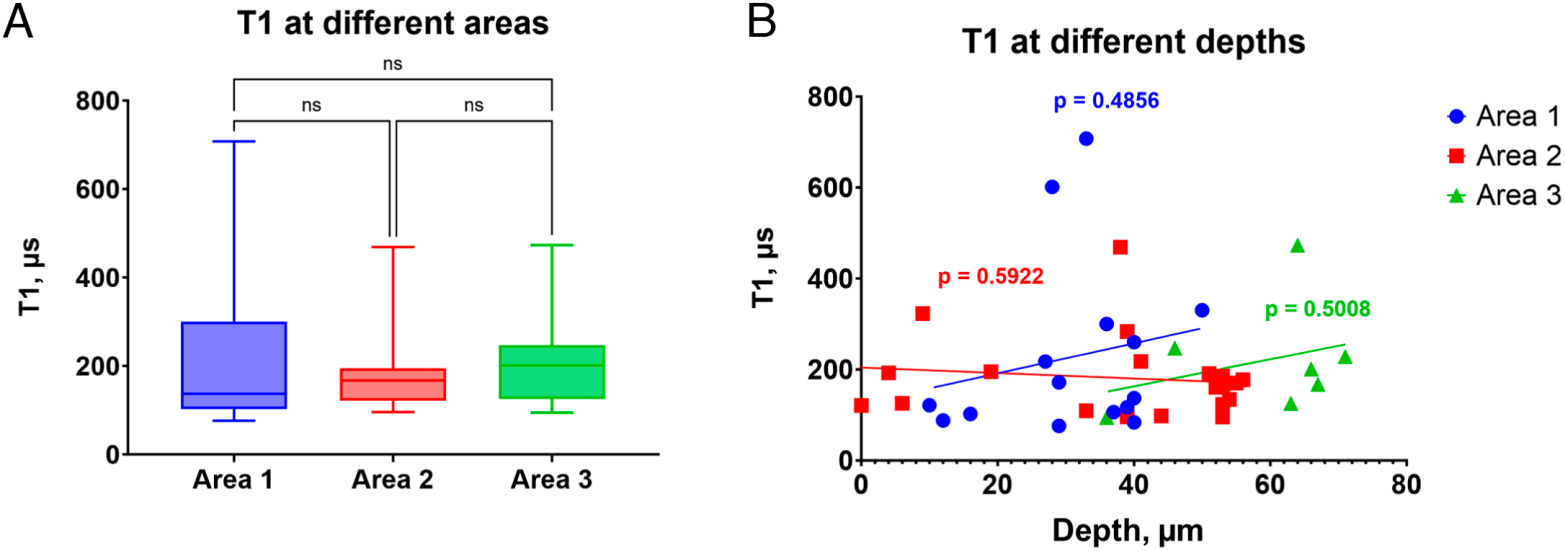
T1 measurements at different locations in a single liver slice. (*A*) A total of 42 measurements on different FNDs located within 3 randomly chosen areas of 50 × 50 µm show no statistically significant differences between the locations. (*B*) Linear regression shows no significant relationship between the T1 values and the depth within the liver slice (measured from the bottom of the slice). *P*-values indicate the significance of the slope deviation from zero. Depth is recorded from the surface of the slice and is consistent between three locations.

While the liver is a relatively homogeneously structured tissue, there might still be differences in the tissue composition of specific areas. These differences occur due to concentration differences in enzymes/proteins involved in production of radicals as well as scavenging radicals. Additionally, there might be location-specific artifacts that occur in T1 measurement. This could be the case if a specific area, for example, the surface vs. the core of the tissue, is better or worse supplied with nutrients. It is also possible that measurements from deep in the tissue are more affected by background fluorescence. To examine the possible effect of such artifacts, we compared T1 measurements in different, randomly chosen areas of the tissue as well as across different depths. As shown in Fig. 6, we did not find any significant differences in T1 in different areas as well as across the reachable depth range (approximately 75 µm). It is worth mentioning that in the subsequent measurements in this article, we excluded the very surface of the tissue on purpose to avoid dead cells from cutting.

### T1 Changes during Exposure to Ethanol or L-Ascorbic Acid.

**Fig. 7.**
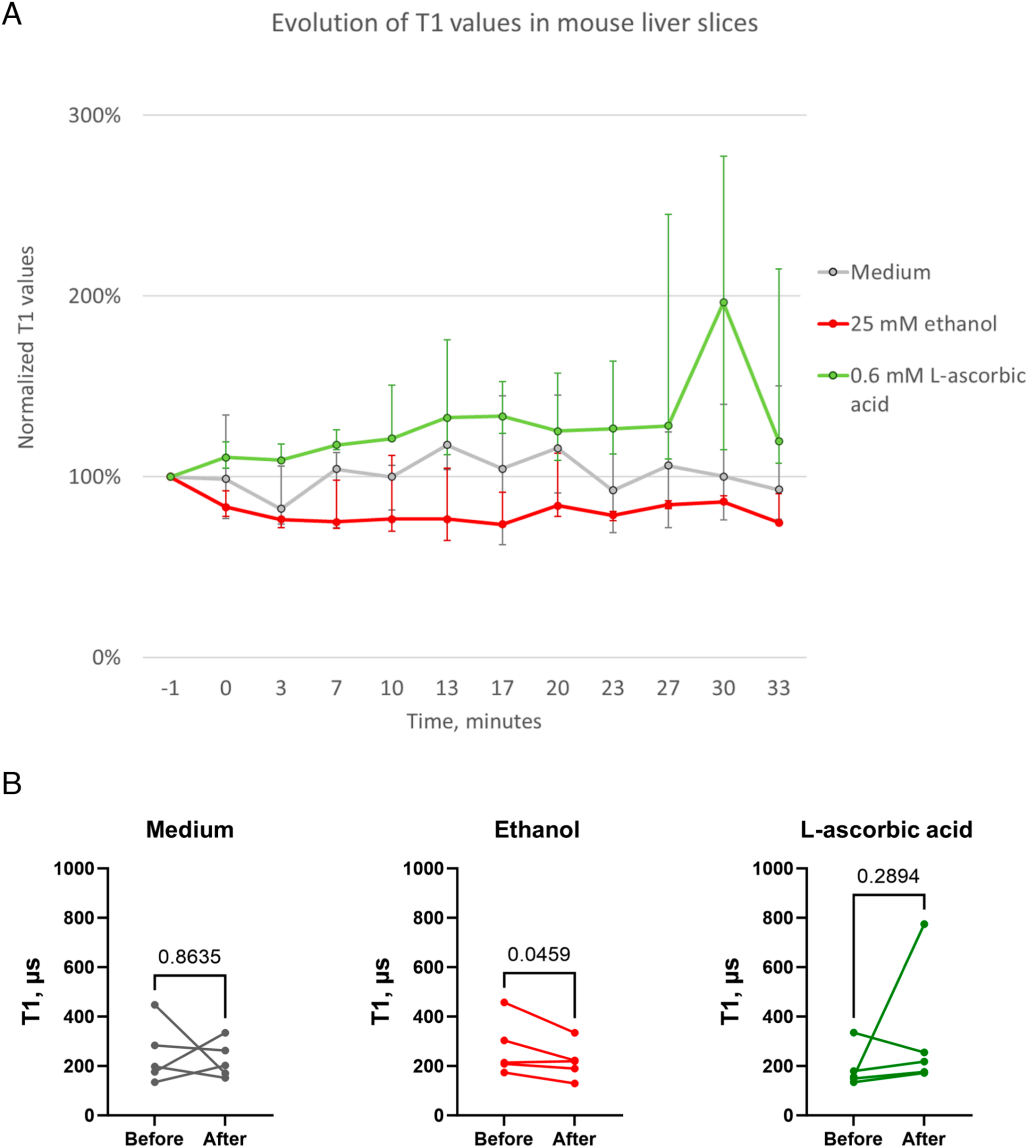
T1 evolution in liver slices exposed to different triggers. (*A*) 25 mM ethanol (red) results in a rapid drop in T1 values recorded from the FNDs in the liver slices, whereas 0.6 mM L-ascorbic acid (green) has the opposite effect. The baseline timepoint is indicated as “−1” min. All T1 values are normalized to the corresponding baseline T1 values, recorded from the same FNDs. Points show the medians of five independent measurements on different liver slices. Error bars indicate the interquartile range. (*B*) Pairwise comparison of the baseline T1 values (“Before”) and final T1 values (“After”) for each experimental setup. Ethanol results in lower T1 values after 33 min of incubation, whereas L-ascorbic acid treatment produces generally higher T1 values, although not reaching statistical significance. Medium (the negative control) does not have a clear effect on the recorded T1 values.

Finally, we performed T1 measurements where we followed a specific FND and then performed an intervention to induce or reduce a stress response (Fig. 7). To induce a stress response, we chose exposure to ethanol due to the well-known link between oxidative stress in the liver and ethanol (32, 53, 54). To reduce oxidative stress, we used L-ascorbic acid (also known as vitamin C), which has already been used earlier to reduce oxidative stress in liver tissue (55). The proof-of-principle experiments are shown in Fig. 7. In line with our expectation, we observed a decrease in T1 and thus an increase in free radical generation after inducing oxidative stress with ethanol. This is similar to what we have observed in cells for other stressors before (15, 16). On the other hand, if we simply replace the medium, we do not observe a significant change in T1 over the measurement time. The relatively small difference between these two conditions might be explained by the fact that the tissue slices are already experiencing a mild stress response due to the measurement conditions. However, we did not observe any decline in T1 from the deterioration of the tissue alone. When we supplied L-ascorbic acid, we observed an increase in T1 which indicates that the radical load was decreased. This is also expected from previous findings in cells where chemicals that inhibit free radical generation or antioxidants led to an increase in T1 (16).

**Fig. 8.**
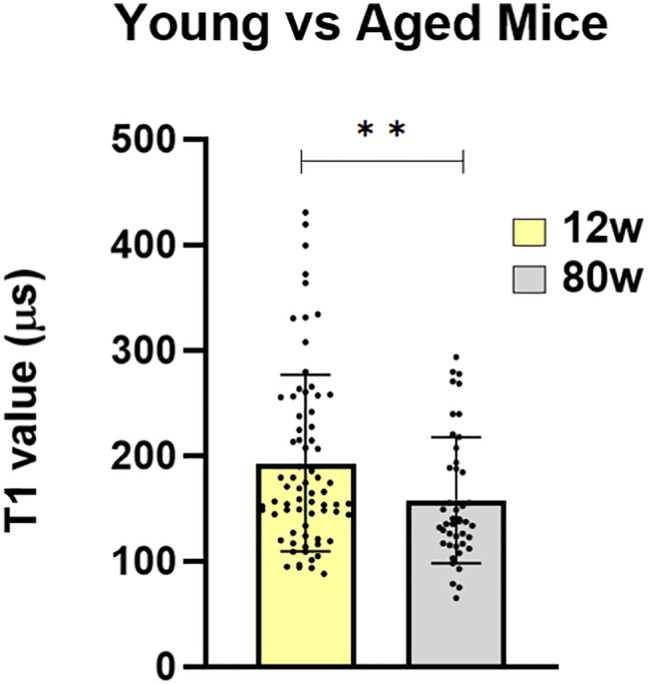
Free radical load in young vs. old mice. We compared the radical load by conducting T1 measurements in PCLS from 12 wk and 80 wk old mice.

As an additional pilot application, we observed the free radical load in PCLS of young (12 wk) and old (80 wk) mice. Fig. 8 shows that the radical load in liver slices from old mice is significantly higher than the one in young mice. Given the increased level of oxidative stress in aged animals, this result was expected (19, 56). However, it should be noted that there are a few differences between our technique and conventional assays.

While it is in principle also possible to measure stress responses in liver tissues with other assays (for example, based on organic probes sensitive to reactive species), our technique has a few distinct advantages. First, nanodiamond relaxometry is specifically sensitive to free radicals—i.e., molecules having at least one unpaired electron (such as hydroxyl radical •OH, or superoxide radical •O^−^_2_). In contrast, most organic dyes detect reactive species in general, including nonradical ones, such as hydrogen peroxide H_2_O_2_. Second, the signal from organic probes reflects the cumulative history of the sample, whereas our technique reports the current free radical load. As demonstrated in the experiments with L-ascorbic acid, we are able to detect both increase and decrease in the amount of free radicals in the sample upon intervention. The organic probes are usually irreversibly changed by the analytes, which makes it more difficult to detect the decrease in free radical load. Third, since we can do a continuous measurement before and after an intervention, it is possible to differentiate the variability between cells and changes caused by the intervention. Fourth, high autofluorescence of the tissues severely interferes with most organic assays, whereas nanodiamond relaxometry can still be successfully performed with correct filter settings.

## Conclusions

By optimizing the measurement protocol, we were able to overcome most of the challenges given by living tissues in performing diamond-based quantum sensing measurements. More specifically, we were able to reduce the background and tissue damage to an acceptable level. Using T1 measurements, we were able to detect free radical generation in living tissues. Additionally, we could follow an increase or decrease in radical concentration in real-time. The major shortcoming of the study is that measurements are limited to the location of FNDs. Additionally, in the current setup, we do not have the possibility to measure under high oxygen content and shaking which limits the measurement time. As a result, it is necessary to perform control measurements to avoid artifacts from stress caused by the measurement condition.

## Supplementary Material

Appendix 01 (PDF)

Movie S1. 3D two-photon microscopy. The movie shows the 3D two-photon microscopy results from different views

Movie S2. Location of FNDs in tissues. The movie shows a tomographic slice + 3D rendered FND volume reconstruction from electron microscopy.

## Data Availability

Raw data for this article can be found in Zenodo (57, 58). All other data are included in the article and/or supporting information.
