# Supplementary material for: Free radical detection in precision-cut mouse liver slices with diamond-based quantum sensing
Source: Proc Natl Acad Sci U S A. 2024 Oct 14;121(43):e2317921121. doi: 10.1073/pnas.2317921121 (PMC11513939; doi:10.1073/pnas.2317921121)
Supplement: Supplementary file 1 — Appendix 01 (PDF) [file pnas.2317921121.sapp.pdf]

Supplementary information

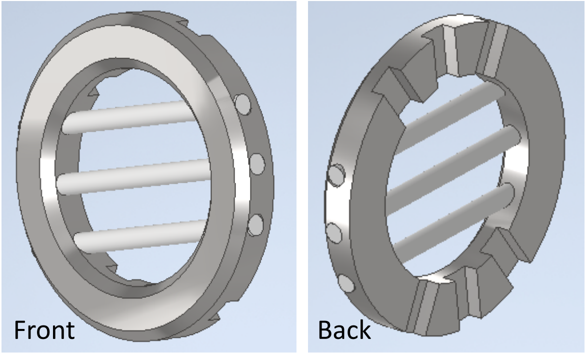

Figure S1. Design of stainless steel anchors for liver slices immobilization.

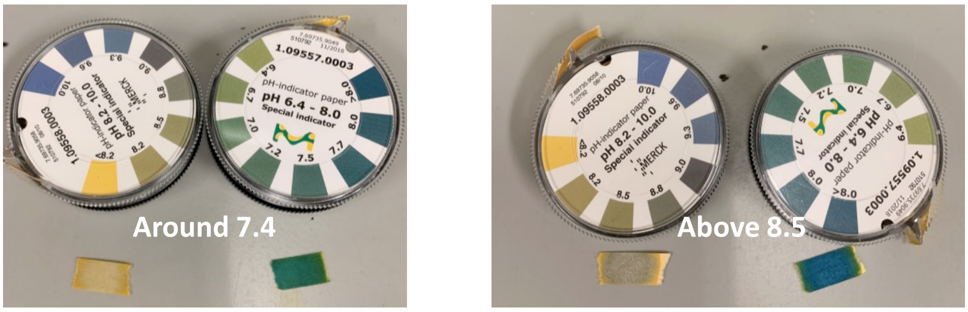

	WEGG	WEGG
standard	37C	RT
	80% O_2/5% CO_2	ambient environment

Figure S2. pH change of WEGG medium in different conditions. Left, standard condition in the incubator. Right, ambient environment at room temperature.

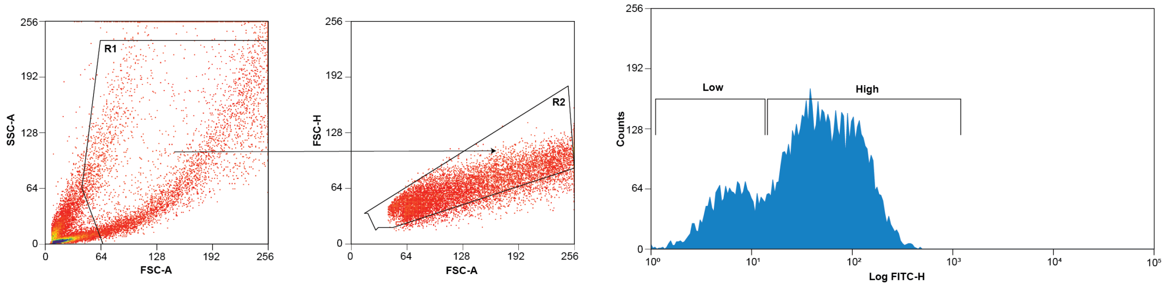

Figure S3. Gating strategy for FACS sorting. Cells were sorted based on their fluorescent counts as well as scattering. Here FSC means forward scatter along the direction of the laser and SSC means scattering at a ninety-degree angle relative to the laser. FITC-h means the autofluorescence in green.

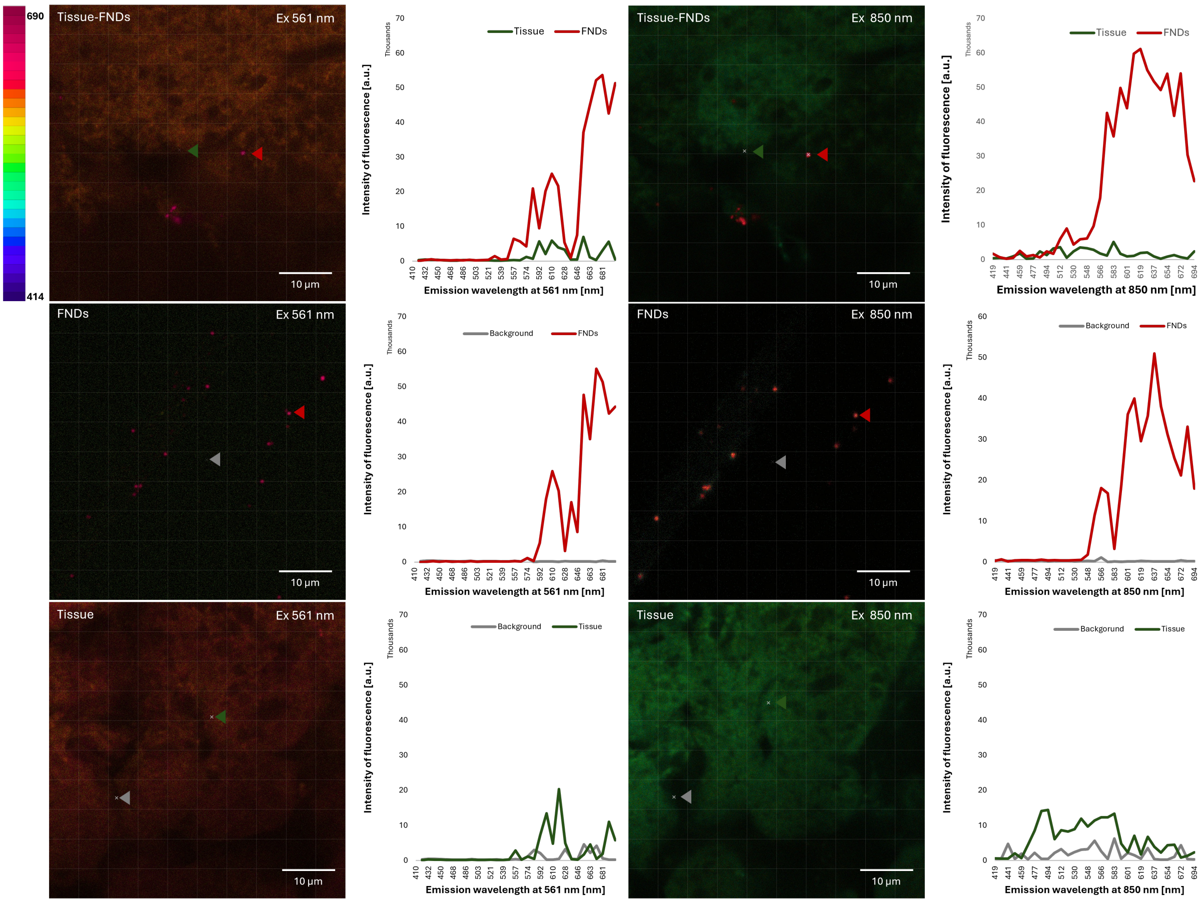

Figure S4. Spectroscopic evaluation of our samples. We investigated the spectra at locations where FNDs are in samples with FNDs only and FNDs within the tissue. We also included tissue without any FNDs as a control.

Supporting movies

Movie S1: 3D two-photon microscopy. The movie shows the 3D two-photon microscopy results from different views

Movie S2: Location of FNDs in tissues. The movie shows a tomographic slice + 3D rendered FND volume reconstruction from electron microscopy.
